# Comparison of Relative Waist Circumference between Asian Indian and US Adults

**DOI:** 10.1155/2014/461956

**Published:** 2014-09-21

**Authors:** Harpreet S. Bajaj, Mark A. Pereira, Rajit Mohan Anjana, Raj Deepa, Viswanathan Mohan, Noel T. Mueller, Gundu H. R. Rao, Myron D. Gross

**Affiliations:** ^1^STOP Diabetes Foundation, Inc., Brampton, ON, Canada L6S 0C9; ^2^Division of Epidemiology & Community Health, University of Minnesota, 1300 South Second Street, Suite 300, Minneapolis, MN 55454, USA; ^3^Madras Diabetes Research Foundation and Dr. Mohan's Diabetes Specialties Centre, Chennai, Tamil Nadu 600 086, India; ^4^Columbia University, New York, NY 10032, USA; ^5^University of Minnesota, Minneapolis, MN 55454, USA

## Abstract

*Background*. Relative to Europeans, Asian Indians have higher rates of type 2 diabetes and cardiovascular disease. Whether differences in body composition may underlie these population differences remains unclear. * Methods*. We compared directly measured anthropometric data from the Chennai Urban Rural Epidemiology Study (CURES) survey of southern Indians (I) with those from three US ethnic groups (C: Caucasians, A: African Americans, and M: Mexican Americans) from NHANES III (Third National Health and Nutrition Examination Survey). A total of 15,733 subjects from CURES and 5,975 from NHANES III met inclusion criteria (age 20–39, no known diabetes). * Results*. Asian Indian men and women had substantially lower body mass index, waist circumference, hip circumference, waist-to-hip ratio, and body surface area relative to US groups (*P* values <0.0001). In contrast, the mean (±se) waist-weight ratio was significantly higher (*P* < 0.001) in I (men 1.35 ± 0.002 and women 1.45 ± 0.002) than in all the US groups (1.09, 1.21, and 1.14 in A, M, and C men; 1.23, 1.33, and 1.26 in A, M, and C women (se ranged from 0.005 to 0.006)). * Conclusions*. Compared to the US, the waist-weight ratio is significantly higher in men and women from Chennai, India. These results support the hypothesis that Southeast Asian Indians are particularly predisposed toward central adiposity.

## 1. Introduction

The number of people with type 2 diabetes in India is highest in the world and is predicted to increase 150% by year 2025, when the projected 69.9 million cases will comprise almost a quarter of the world's diabetic population [1]. The Asian Indian (AI) phenotype, which refers to certain unique clinical and biochemical characteristics such as greater abdominal obesity despite lower body mass index, has been well documented and shown to make AI more prone to diabetes [2–14] and coronary artery disease [15–19] than Caucasians (C) of European ancestry.

The World Health Organization has addressed this paradox of low obesity and high chronic disease risk in Asian populations by setting lower thresholds of BMI to identify those who may be at high risk [20]. However, some have suggested that BMI has a relatively weak association with chronic disease prevalence [21, 22] and with visceral abdominal fat [23] in AI. As alternative measures, studies in various populations, including AI [9, 22–24], US [25], and elsewhere [26], suggest that either waist circumference (WC) alone [23, 25] or waist-hip ratio (WHR) [9, 22, 24, 26] may be a better single anthropometric marker of chronic disease risk, as compared to BMI, because they may more specifically reflect abdominal body fatness. However, WC alone does not reflect lean body mass, which is known to be protective [27], and fails to allow comparisons between subjects and populations due to confounding by body size and weight. Evidence that high WHR may contribute to the high incidence of diabetes in AI is equivocal [19, 28–30].

To address the potential influence on chronic disease risk of high abdominal fatness relative to total body fatness in AI, we propose waist-to-weight ratio (WWR), as a single continuous index, to distinguish differences between cultures in the propensity to store fat in the visceral depots, consistent with differences in insulin resistance, diabetes, and cardiovascular disease (CVD). Therefore, we hypothesized that the WWR will be higher in Indian men and women than in US men and women. This hypothesis was tested by comparing anthropometric characteristics between young adults of the National Health and Nutrition Examination Survey (NHANES III) [27] to young adults of the Chennai Urban Rural Epidemiology Study (CURES) [31].

## 2. Subjects and Methods

### 2.1. Study Populations

NHANES III [27] is a large cohort representative of the US population, with minority groups oversampled. It was conducted by the National Center For Health Statistics and the Center for Disease Control and Prevention on a nationwide probability sample of approximately 33,994 persons aged 2 months and over from mid-1988 to mid-1994. The cross-sectional survey was designed to obtain nationally representative information on the health and nutritional status of the US population through interviews and direct physical examinations. Written informed consent was obtained from all participants and the National Center for Health Statistics approved the protocol. Full details of the study design, recruitment, and procedures are available from the US Department of Health And Human Services [31].

CURES [31] is a large cross-sectional field survey of representative samples of the area in and around Chennai, the largest city in south India and the fourth largest city in India. This study recruited 26,001 subjects who were a random sample of the population of Chennai (representing the urban component) and villages around Chennai (representing the rural component). The study commenced in August 2001 with the objective of comparing the prevalence of various components of Insulin Resistance Syndrome and various diabetes related complications. Ethical committee approval was obtained prior to the start of the study and an informed consent was obtained from all the study subjects. Details on the study design, recruitment, and phases of the survey are published elsewhere [31].

### 2.2. Anthropometric Assessments

In NHANES III, height was measured to the nearest 0.1 cm with calibrated stadiometer, without shoes. Weight was measured to the nearest 0.1 kg with calibrated scale, allowing light clothing. WC was measured to the nearest cm with tape measure at the highest point on the iliac crest, while the subject was at minimum respiration, allowing light clothing. HC was measured to the nearest cm with a tape measure at maximum extension of the buttocks, allowing light clothing. In CURES, height was measured to the nearest cm with a tape measure, subjects standing upright without shoes. Weight was measured to the nearest 0.5 kg with a calibrated scale, allowing light clothing. Waist was measured to the nearest cm with a tape measure at the smallest horizontal girth between the costal margins and the iliac crest at minimal respiration. Hip was taken as the greatest circumference at the level of greater trochanters (the widest portion of the hip) on both sides. It was measured to the nearest cm with a tape measure. In order to compare the sex-specific prevalence of overweight and obesity among the four ethnic groups, we used the currently accepted definitions based on BMI and WC cutoffs [33]. These include lower cutoffs for AI recommended by WHO for BMI [25] and IDF for WC [34].

### 2.3. Exclusion Criteria and Final Sample Sizes

In order to minimize the likelihood of bias due to age-cohort effects and the potential impact of clinical or subclinical illness on anthropometry, we implemented the following exclusion criteria: (1) missing or aberrant values for anthropometric variables (excluded 7,824 from NHANES III and 446 from CURES), (2) missing race/ethnicity or race/ethnicity other than African American, Caucasian, or Mexican American (excluded additional 1,130 from NHANES III), (3) age < 20 or >39 (excluded additional 18,735 from NHANES III and 9,270 from CURES), (4) blood sugar < 50 mg/dL or >200 or known diabetes (excluded additional 329 from NHANES III and 462 from CURES), and (5) BMI < 14 kg/m^2^ (excluded none from NHANES III and additional 93 from CURES). There were a total of 21,705 subjects (5,976 from NHANES III and 15,729 from CURES) who met the inclusion criteria.

### 2.4. Statistical Methods

All analyses were sex-stratified and performed using SAS version 9.1 (Cary, NC). We compared the unadjusted prevalence of overweight and obesity, based on BMI and waist categories, between AI and the three US race groups using chi-square analysis. General linear regression models were used to estimate unadjusted and adjusted least squares means (±se) of the anthropometric variables (dependent) by race and sex group. All *P* values are 2-sided. Estimates were not weighted according to the NHANES sampling scheme because our aim was to make comparisons to the CURES population sample and not to make estimations for the entire US population.

## 3. Results

Race- and sex-stratified sample sizes and unadjusted anthropometric characteristics are shown in Table 1. Briefly, Asian Indian men and women had substantially lower height, weight, body mass index (BMI), WC, hip circumference (HC), and body surface area relative to all US ethnic groups (all sex-specific *P* values <0.0001). Asian Indian men had a lower mean waist-to-hip ratio compared to the Caucasian and Mexican American men (*P* < 0.0001) but slightly larger mean waist-to-hip ratio than the African American men (*P* = 0.03). Asian Indian women had a higher mean waist-to-hip ratio compared to the African American and Caucasian women (*P* < 0.0001) but similar to the Mexican American women (*P* = 0.43).

### 3.1. Ethnic Variations in BMI and WC Categories

**Figure 1:**
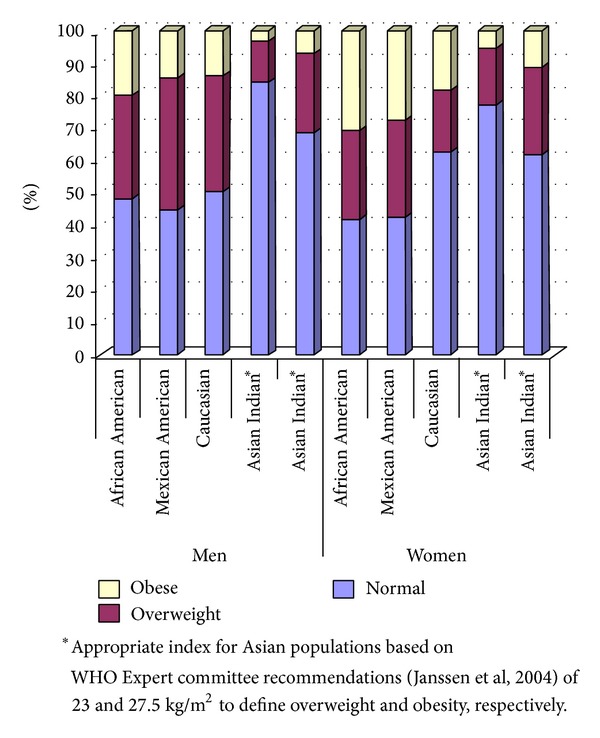
BMI categories stratified by ethnicity and sex.

**Figure 2:**
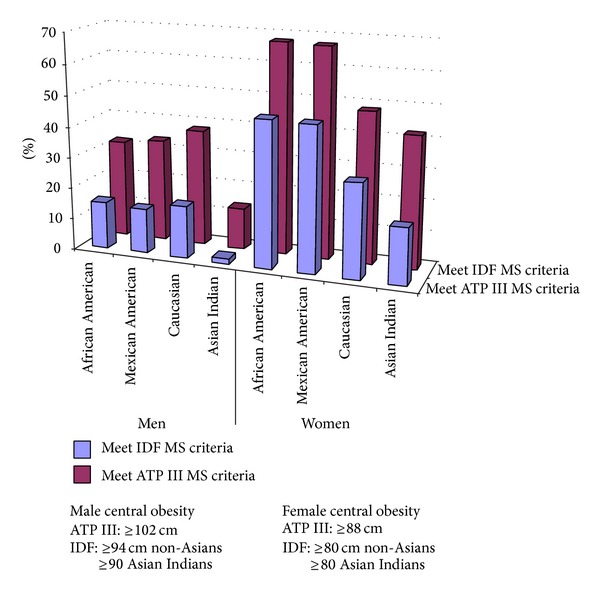
Waist categories stratified by ethnicity and sex.

In men and women alike, as shown in Figure 1 based on BMI criteria, the frequency of AI in the normal weight category (84.2% for men, 77.4% for women) was significantly greater, and the frequency of overweight and obesity was lower, than any other ethnic group (*P* < 0.0001 for all sex-stratified comparisons). This remained true even after applying lower cutoffs for the Asians Indians as suggested by the WHO Expert Committee recommendations [25], except for the frequency of overweight Asian Indian women which was similar to the African American women (*P* value = 0.69), slightly less than Mexican American women (*P* value = 0.03) and significantly less than Caucasian women (*P* value <0.0001). In Figure 2, it is seen that the Asian Indian men and women have lower central obesity meeting NCEP's metabolic syndrome cutoff values for WC than all other groups (*P* < 0.0001). When applying the ethnicity driven IDF cutoffs, the differences between the AI and other groups were attenuated but remained statistically significant (*P* < 0.0001), even with the lower cutoffs suggested for the Asian Indian men.

### 3.2. Correlations among Anthropometric Variables

**Figure 3:**
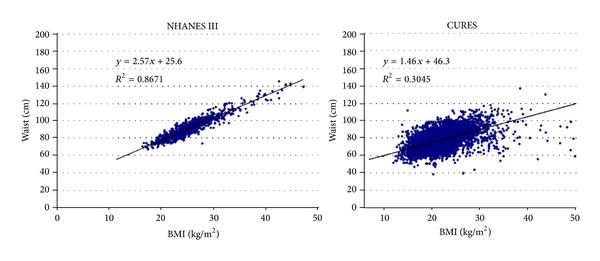
Correlations of waist circumference and BMI in US Caucasian and Asian Indian men. The corresponding *R* ^2^ value for women of NHANES III was 0.85, in comparison to 0.31 for CURES women. The *R* ^2^ across the other two NHANES III race/ethnic groups for men and women ranged from 0.81 to 0.88.

Table 2 (men) and Table 3 (women) include the unadjusted sex- and population-specific Pearson correlations among the various anthropometric variables. We pooled the US race groups within sex because the correlations did not vary in important ways among US race groups. The magnitudes of these correlations were all lower, without exception, in the AI compared to the US, with most of the Asian-US differences being large. For example, the magnitude of the correlations between waist and either body weight or BMI ranged from 0.56 to 0.63 in Chennai men and women, compared to 0.90 to 0.93 in US men and women. The scattergrams in Figure 3, shown for men, demonstrate the considerable population differences in the association between waist and BMI between Chennai and US Caucasian men. For any given BMI in AI there was considerably more interindividual variation in the waist than is observed in the US. Very similar results were observed for women and for the other US ethnic groups (data not shown).

Of note, age and height adjustment did not alter the correlations in magnitude or direction in any considerable manner (data not presented). In contrast, as presented in Tables 2 and 3, weight adjustment altered correlations between hip and waist as well as between hip and WHR among the US population. The strong correlations between hip and waist were considerably attenuated by adjusting for weight in both genders in the US population, whereas this adjustment had little effect in the Asian Indian population (Tables 2 and 3). Of particular note the different direction of the correlation between hips and WHR for AI (−0.12 for men, −0.23 for women) was compared to US (0.40 for men, 0.26 for women). However, in the US the correlation between hips and WHR was confounded by the high correlation between waist and weight. As such, further adjustment for weight reversed the direction of this correlation in the US (−0.37 for men, −0.53 for women). Weight adjustment marginally strengthened the magnitude of the correlation in the AI, but because the correlation between waist and weight is relatively low, the direction of the association remained inversed (−0.29 in men, −0.36 in women).

### 3.3. The Waist-to-Weight Ratio (WWR)

To further evaluate possible differences between populations in central adiposity while taking into account differences in total body mass, we computed the WC to body weight ratio. The WWR followed a normal distribution for all sex and population groups. The median WWR was highest among men and women from India and was in fact higher than the 75th percentile of all other groups. The means of WWR before and after adjustment for height are shown in Table 4. As can be seen, WWR was significantly higher (*P* < 0.0001) in the AI than in US for both sexes. The attenuation of these differences by age and height adjustment was minimal.

## 4. Discussion

The present study compares a number of traditional anthropometric factors between young adults of the US representative NHANES III survey, including three race/ethnic groups, and the population-based CURES survey of AI. Our observations confirm the findings of others that the AI tend to be much smaller than the US population in all traditional anthropometric measurements—body weight, body height, BMI, body surface area, hips, and WC. In contrast, we observed the WHR, which some studies have found to be higher in AI than in other groups [22, 24, 26], to be somewhat higher in AI women compared to US women, but not different, or in fact lower, among AI men compared to US men. In our analyses, the differences between means of WHR, though significantly higher in AI women compared to Caucasian and African American women, were small.

It is possible, therefore, that if AI have a propensity towards excess accumulation of visceral body fat, it is being masked, perhaps especially in the men, by large differences in overall body size between populations. Based on the findings of the present study, it appears that our novel measurement, WWR, is a simple anthropometric index capturing population differences in propensity for intra-abdominal fat storage. The WWR would appear to sufficiently account for the large confounding influence of overall body size and general adiposity. For any given body weight, larger WC could reflect larger intra-abdominal fat depots. Individuals with relatively high body weights who tend to carry much of their weight in muscle and subcutaneous fat depots peripherally would be expected to have lower WWR and lower risk for diabetes and CVD, hypothetically. Based on our observations, the distribution of WWR in young adults from Chennai, India, is considerably shifted to the right in comparison to similarly aged adults from three race/ethnic groups of the US population.

An intriguing observation from comparisons performed in present study was the vastly different correlation between WC and BMI between young adults of Chennai, India, compared to those from three US race/ethnic groups. In the US over 80% of the variation (*r* =  ~0.9) in WC is explained by BMI, whereas in Chennai, India, these two parameters shared less than 40% common variance (*r* =  ~0.60). This observation helps us to understand the basis for the higher WWR in Chennai than in US. At any given BMI there is clearly much more variation in the WC of Asian Indian young adults than in US young adults. Indeed, the variance of WWR in the Chennai men and women was threefold higher than that of the US men and women (0.03 versus 0.01). This observation suggests that AI may have a heightened propensity for accumulating central adiposity, for any given body size, than other populations.

A strength of the WWR is that it retains simplicity in measurement and calculation, making it desirable over WHR and BMI in this regard, and allows comparisons between people and populations varying in overall body size, making it more useful than the WC alone. A possible limitation of the WWR is that it does not consider height. To look further into the usefulness of height, we did compute (data not presented) a waist-to-BMI ratio (WBR). This ratio differentiated the two populations on an ecological level in a similar manner as did the WWR. Using such ratio did not seem to provide any added benefit to the WWR, while it may be fraught with a number of conceptual and statistical problems discussed by others [35, 36]. Another conceivable limitation of the WWR is that the denominator (weight) is highly correlated with the numerator in many populations (see Tables 2 and 3).

Strengths of the present study are multiple. It is a population-based and a large-scale study of AI who have a high risk of diabetes; the study compares them with the three main ethnic groups of the US NHANES population. The high quality standardization of anthropometric measurements in both cohorts minimizes measurement errors and biases. Despite several practical advantages of ecologic studies, including low cost, there exist several methodological limitations to any ecological study in its ability to make causal inferences [32]. Ecological studies often suffer from biases (ecological fallacy or aggregation bias), which represents the failure of expected ecological effect estimates to reflect the effect at the individual level. A potential strategy suggested for minimizing ecological bias is to use smaller units in order to make the groups more homogeneous with respect to the exposure [32]. For this reason, we included only nondiabetic young adults who were not underweight, which also helps to minimize the effects of temporal ambiguity (presence of disease effecting body habitus) and different cohort effects between populations. Possible limitations of this study originating from the somewhat different anthropometric measurement standards were described in the methods section. Given that these differences were small, we believe that the results are not materially biased by misclassification due to such measurement differences. The fact that the CURES survey was conducted in and around the southern Indian city of Chennai begs the question of whether these results can be generalized to the whole Asian Indian population.

We believe that our findings in this study are provocative and should stimulate further research into novel anthropometric features that may differentiate populations at very high risk for chronic disease, despite relatively low adiposity. The findings should motivate future coordinated research on complex exposures, their social and behavioral determinants, and possible interventions. Certainly, the WWR should be validated using more precise clinical methods of measuring fat depots, including DEXA and CT scan. The WWR should be investigated in other studies in order to address possible influences of age, ethnicity, gender and nutritional status, and its relation to chronic diseases and mortality.

## Figures and Tables

**Table 1:** Mean (sd) anthropometric variables by population.

	Male				Female			
	African American	Mexican American	Caucasian	Asian Indian	African American	Mexican American	Caucasian	Asian Indian
*N*	884	1043	799	7577	1131	1098	1021	8152
Height (cm)	176.9 (7.13)	170.0 (6.51)	177.5 (6.68)	164.7 (7.63)	163.6 (6.42)	157.4 (6.10)	164.0 (6.51)	156.3 (7.05)
Weight (kg)	82.5 (19.12)	75.4 (13.91)	81.3 (16.64)	59.2 (10.29)	74.5 (19.68)	67.3 (16.01)	67.3 (16.97)	54.7 (10.18)
BMI (kg/m^2^)	26.3 (5.56)	26.0 (4.30)	25.7 (4.70)	21.8 (3.80)	27.8 (7.04)	27.1 (6.15)	25.0 (6.08)	22.4 (4.45)
Waist (cm)	88.7 (14.54)	90.2 (11.17)	91.7 (12.71)	78.5 (10.10)	89.3 (16.20)	88.0 (14.29)	83.5 (14.86)	77.6 (10.59)
Hip (cm)	99.3 (11.13)	96.4 (7.84)	99.0 (8.86)	87.8 (9.74)	104.6 (13.82)	101.3 (12.38)	100.5 (12.61)	89.6 (11.33)
Waist/hip	0.89 (0.07)	0.93 (0.06)	0.92 (0.06)	0.90 (0.07)	0.85 (0.08)	0.87 (0.07)	0.83 (0.08)	0.87 (0.08)
BSA [32]	2.00 (0.24)	1.88 (0.19)	1.99 (0.22)	1.64 (0.16)	1.83 (0.25)	1.70 (0.21)	1.74 (0.22)	1.53 (0.15)

**Table 2:** Pearson correlations—men.

	WAIST		HIP		WHR		WT		BMI		BSA		
	CURES	NHANES	CURES	NHANES	CURES	NHANES	CURES	NHANES	CURES	NHANES	CURES		NHANES
WAIST			**0.72**	**0.18**	**0.42**	**0.84**	**0.60**	**0.90**	**−0.05**	**0.20**	**0.10**	**0.19**	
HIP	0.82	0.89			**−0.29**	**−0.37**	**0.54**	**0.95**	**−0.01**	**0.20**	**0.05**	**0.20**	
WHR	0.45	0.77	−0.12	0.40			**0.18**	**0.48**	**−0.06**	**0.09**	**0.09**	**0.15**	
WT	0.61	0.90	0.57	0.95	0.18	0.48			**0.83**	**0.91**	**0.97**	**0.99**	
BMI	0.56	0.93	0.49	0.90	0.20	0.60	0.82	0.91			**−0.03**	**0.36**	
BSA	0.57	0.84	0.54	0.92	0.16	0.41	0.97	0.98	0.66	0.82			
HT	0.09	0.18	0.13	0.35	−0.03	−0.12	0.32	0.45	−0.26	0.05	0.53	0.61	

Not bold: unadjusted.

Bold: adjusted for age, height, and weight.

WAIST: waist circumference; HIP: hip circumference; WHR: waist-to-hip ratio; WT: body weight; BMI: body mass index; BSA: body surface area; HT: height; CURES: Chennai Urban Rural Epidemiology Study; NHANES: National Health and Nutrition Examination Survey.

**Table 3:** Pearson correlations—women.

	WAIST		HIP		WHR		WT		BMI		BSA	
	CURES	NHANES	CURES	NHANES	CURES	NHANES	CURES	NHANES	CURES	NHANES	CURES	NHANES
WAIST			**0.65**	**−0.002**	**0.45**	**0.83**	**0.60**	**0.90**	**0.01**	**0.15**	**0.12**	**0.19**
HIP	0.79	0.87			**−0.36**	**−0.53**	**0.58**	**0.94**	**−0.02**	**0.23**	**0.07**	**0.10**
WHR	0.40	0.69	−0.23	0.26			**0.10**	**0.40**	**0.04**	**0.01**	**0.07**	**0.17**
WT	0.63	0.90	0.62	0.94	0.09	0.40			**0.86**	**0.94**	**0.97**	**0.99**
BMI	0.57	0.92	0.60	0.93	0.04	0.46	0.86	0.94			**−0.10**	**0.23**
BSA	0.60	0.86	0.57	0.91	0.12	0.37	0.97	0.98	0.72	0.87		
HT	0.06	0.07	−0.01	0.17	0.11	−0.11	0.20	0.29	−0.31	−0.05	0.42	0.44

Not bold: unadjusted.

Bold: adjusted for age, height, and weight.

WAIST: waist circumference; HIP: hip circumference; WHR: waist-to-hip ratio; WT: body weight; BMI: body mass index; BSA: body surface area; HT: height; CURES: Chennai Urban Rural Epidemiology Study; NHANES: National Health and Nutrition Examination Survey.

**Table 4:** Mean (se) of waist-to-weight ratio by sex and population.

	Male				Female			
	African American	Mexican American	Caucasian	Asian Indian	African American	Mexican American	Caucasian	Asian Indian
Waist/weight	1.09 (0.006)	1.21 (0.006)	1.14 (0.006)	1.35∗ (0.002)	1.23 (0.005)	1.33 (0.005)	1.27 (0.006)	1.45∗ (0.002)
Waist/weight	1.21 (0.006)	1.27 (0.005)	1.26 (0.007)	1.37∗ (0.002)	1.24 (0.005)	1.30 (0.005)	1.28 (0.005)	1.40∗ (0.002)
adjusted for age and height								

*P < 0.0001 compared to all other groups.

*P* < 0.05 for all other sex-specific comparisons, except for Mexican American men and Caucasian men in the age and height-adjusted model.
